# Some Observations in the Course of the Life of an Active M. D.

**Published:** 1913-04

**Authors:** A. F. Sampson

**Affiliations:** San Francisco, Cal.


### TEXAS MEDICAL JOURNAL.

Established July, 1885

F. E. DANIEL, M. D., - - - - - Editor, Publisher and Proprietor

Published Monthly.—Subscription, $1.00 a Year.

VOL. XXIX. AUSTIN, APRIL, 1913. No. 10.

The publisher is not responsible for the views of the contributors.

### Original Articles.

For Texas Medical Journal.

### Some Observations in the Course of the Life of an Active M. D.

BY A. F. SAMPSON, M. D., SAN FRANCISCO, CAL.

"Old Nature is a kindly dame,
And keeps her plenty on the shelf;
But she will yet assert her claim,
In due time to protect herself.
Outraged, she grows terrific; then
Wreaks her vengeance many fold;
You may coax her back into her den,
But you cannot bribe her off with gold."

The aptness of these lines has so often manifested itself in the course of my professional observations, that I deem it to be a text worthy of the attention of all.

As a member of the regular school of medicine, and a graduate of the medical department of the University of Virgina, where honor and integrity have ever been among the leading virtues, I have always upheld as a standard the saying that the true physician who worthily practices his profession stands next to God.

The maxim "Humanum est errare" is so potential in the rugged and devious byways of life through which we travel, and the failure properly to observe it is so responsible for the major part of the diseases which physicians are called upon to combat, that I cannot refrain from making it a basis for the thoughts which I shall here present.

As my observations ripened, I became more and more convinced that the great majority of diseases arise from one or more of the many infections which so abundantly prevail. To such extent has this belief impressed me that I have formed the belief, and given expression thereto, that the day is not far distant when, in our professional duties, it will be of the highest importance for us to devote our best energies to preventing the sources of infection, and that, therefore, our most important work for the relief of diseases must be along the lines of preventive medicine.

This belief has, in recent years, obtained a substantial foothold in the profession, as has been manifested by the energetic way in which the self-sacrificing members of the medical profession have developed their work in accordance with such belief.

It is depressing and saddening to contemplate the commercialism and many other repellant "isms" and "cults" that have fastened themselves upon the community generally as well as upon the noble profession of medicine.

In what direction must we seek the cause of this unfortunate condition?

It is of course obvious that, both in the physical and mental sense, a physician is confronted with trying conditions in administering to the wants of the sick. To a large extent the blame for this condition is to be attributed to the profession itself. There has been a lack of candor towards the patient, who, in his weakened condition, has the right to demand that his physician shall disclose clearly and in the open his knowledge and procedure. Why should not efficient knowledge, based upon energy and integrity, be exhibited openly and frankly in the medical profession as well as in any other pursuit?

The fact that the medical profession has, in general, failed properly to observe these considerations, has been one the prime causes of bringing about undesirable conditions and enabling such conditions to gain a sinister hold upon the community as well as upon the profession itself.

A line of cleavage has thus been drawn, with resulting injury in many directions. This must continue until the above-mentioned errors are corrected. In the past men of integrity and energy have labored to uphold the standard of the medical profession.

I quote herewith extracts from an able newspaper editorial entitled "Is the Doctor His Worst Enemy?" The editorial commented upon a lecture delivered by one of our able teachers of medicine, and asked: "Is the doctor, in his zeal to conquer disease, working to reduce the sum total of medical men?" I answer, "Assuredly, yes, as the medical profession is now more in need of quality than quantity." "The physician who makes it his life work to prevent untold distress to his fellow-man, should be regarded with the highest admiration."

It has been said that the physician is born to his calling. I consider that this applies as well to every man who follows the calling for which he is best fitted and who has had the advantage of environments that tend to develop knowledge, application and integrity.

My career as a physician began in the swamps of Madison Parish, Louisiana. How I groped for knowledge, though the possessor of the diplomas and insignia of the so-called educated man. It was indeed fortunate that I realized how little I did know and how great the necessity for further research. (I will show, later on, that even under such unpromising conditions I succeeded in producing results which had been vainly striven for by some of our scientific men in their efforts to combat disease.)

In the observations of a physician, too great importance cannot be given to the question of nutrition and its disturbances which cause illness. To the physician, this subject is as important, in the solution of his professional problems, as the compass is to the mariner in guiding the course of his vessel. Everyone should consider his body as a precious gift from God, a marvelous piece of mechanism, masterly beyond human description, and so delicate that a word, a breath, a look, nay, a thought, may injure it. The depth and force of this idea, as applied to nutrition and nutritive changes, must impress us all.

When I was a member of the Board of Medical Examiners in Texas, a favorite question of mine was: "What are the physiological functions of the sympathetic nervous system?" Those applying for certificates to practice medicine in that district so seldom touched upon its influence over the functions of nutrition, that it became quite evident that our profession was sadly overlooking the vital subject of nutrition and its various sources of disturbance. We would then discuss the action of the sympathetic nervous system in controlling functions of nutrition, and the result of such discussion would frequently be that both the examiner and the examined would be the gainers thereby.

It is no easy task for the physician to educate the patient on this one point alone. The ordinary man is so strongly imbued with his belief in drugs as all-powerful, that it is difficult to make him understand how much depends upon his personal habits and upon his co-operation with Nature.

Consider how many cases we see suffering from toxaemias, or infections caused by undue regard for the functions of the nervous system or carelessness in properly correcting the early stages of infection. It is necessary that the physician teach his patient to regard pain and discomfort as Nature's danger-signals. Here is one, for instance, who fails to appreciate the effect of undue nervous disturbance: What do we find has taken place? Let us follow one branch of the very perturbed stream of nerve-strain: We find him indulging in an unwholesome menu, masticating improperly, and not considering the hordes of infectious germs that are carried down from the mouth. The food thus improperly taken is dumped into a stomach not prepared to receive it. Now what takes place with such a meal? The proper physiological conditions of the stomach being thus disturbed, the result is stomach indigestion and impaired protection to the stomach, and fermentation and putrefaction soon start within the stomach. Upon the discharge of the contents of the stomach into the bowel tract the trouble is much more aggravated. If such a patient is fortunate enough to vomit and have a diarrhoea, which is Nature's method of emptying this septic tube, a marked advantage is gained. The continuance of these improper conditions of food selection and mastication will ultimately result in injury to the lining walls of the stomach and intestines and also in toxaemias, which are so far-reaching in their deleterious effects upon the system in general. The patient consults the physician for relief from what he deems a mere bilious attack, and does not realize that the "bilious attack" is simply Nature's warning of a disorder that is far more deep-seated.

In a recent conversation with an experienced dentist, the following question was discussed: Is Riggs' disease a result of local disturbances, or does the disease originate in great measure from a source beyond the mouth, namely, a toxaemia, which, by lowering resistance of all the tissues of the body, allows the omnipresent organisms in the mouth to assail the gums? When I considered the poor results that had been attained by local treatment for Riggs' disease, I resolved, if possible, to ascertain just what is the primary cause of this trouble. My observations revealed that the dentist, when cleaning the teeth and making local antiseptic applications, would frequently lay stress upon the presence of too much uric acid in the system and would order the use of milk of magnesia. I desired to know why there was too much uric acid, whence it had arisen, and how we should proceed to correct it. From my consideration of the subject I believed that the condition was due primarily to a toxaemia. I have yet to meet a dentist whose research into the causes of this disease will enable him to satisfactorily answer the all important Why. It is quite evident, in this one branch of dentistry, that either the alliance between the medical and dental professions is not sufficiently close, or commercialism, that many-headed monster, is again lifting one of its heads too prominently.

In commenting as a physician upon some of these minor points, I do so only in the hope that due consideration will be given to the same along the lines of our noble profession.

Several years ago one of our most prominent surgeons asked me to compare the surgeon and the physician. My reply was that Old Nature takes off her hat to the surgeon and says to him, "Do your work properly and I will be your handmaid in restoring the afflicted," whereas she says to the physician, "I take the lead; do your part as my handmaid." Since our profession has made such marvelous progress in overcoming infections, the above expressions as to the status of the surgeon and physician should be considerably modified.

It has been my experience that the great majority of people ignore the early warnings of offended Nature (the causes for which could readily be removed by proper medical treatment), and that their continued indifference to these warnings results in the establishment of conditions that can only be corrected by the surgeon's knife. How regrettable it is that the wonderful accomplishments of good surgery are so misjudged by the vast majority of the laity.

### A FEW OBSERVATIONS ON THE GREAT VALUE OF DIAGNOSIS.

In achieving results in any pursuit, how immensely valuable it is to know just what problems are to be solved.

One of the best diagnostic teachers I ever had the pleasure of knowing created within me a most indelible impression by preceding his clinical investigation with these ringing words: "Now, gentlemen, in this case, what have we here, with its varied disturbances?"

In medical diagnosis, we are dealing with extremely complex problems, especially when we consider the physiological function of nutrition, how very delicately it is adjusted, and how easily offended. Hence, how unfortunate it is that the laity is so prone, upon the most casual glance at a sufferer, to assert a diagnosis and to suggest a "sure-cure." We cannot but realize the great need of educating the people along this line and also the force of the saying, "Oh, what fools these mortals be."

AS TO SPECIALISTS.

From my observations as a member of the medical profession, I am constrained to believe that the work of the specialist leaves much room for improvement. In the first place, we all know how prone every man is to see things only from his own peculiar, and ofttimes narrow, point of view.

(See *Journal of The American Medical Association,* Vol. 80, No. 24, page 2827, wherein, among other things, the following is stated:

"We are facing in this country a situation which reflects discredit on the general medical profession, and particularly on the specialties of the eye, ear, nose and throat. This situation is one that has developed out of our own deplorable lack of proper standards in medical education," etc.)

What part commercialism has played in the work of the specialist I shall leave to the determination of those who are in a position to criticize. I will, however, give one or two instances showing that specializing has too closely narrowed the field of our profession.

A Mr. B. was troubled with disturbances of his vision and consulted some of our leading specialists here, who informed him that the cause of the trouble was a peculiar form of disturbance of the retinal vessels. Inasmuch as the patient's condition did not improve under their treatment, he went East and consulted the best oculists of Johns Hopkins University as well as those of Philadelphia and New York. These oculists confirmed the advice which he had received from the specialists here. He returned home not at all improved and very much discouraged. He was subsequently led to believe that his trouble was probably due to some disturbance in the functions of the sympathetic nervous system in its controlling influence over the vasomotor system, in which disturbances toxaemias play so important a part. Upon examination, it was found that such was the fact and that the eye trouble was primarily caused by a toxaemia. This was properly treated with very satisfactory results.

I mention the following instance in further support of my theory that the oculists are sometimes too narrow in their specializing:

About three years ago, a Mrs. J., an elderly woman, came to me after obtaining glasses from an optician. I referred her to an oculist in order to get his opinion as to the condition of the eye. The oculist reported to me that he had found a choked optic disk and other eye conditions which indicated inter-cranial pressure, and said his diagnosis was probably brain tumor. My clinical knowledge of the patient made me skeptical as to the diagnosis of brain tumor. I told the patient's family what the oculist's opinion was and stated that I did not agree with it. A nerve specialist was then consulted, and, after examination, he stated that he was inclined to confirm the opinion of the oculist. My own opinion was that while there was sufficient pressure to cause the choked disk and congestion of the vessels of the eye, I nevertheless believed the trouble was due in the first instance to vasomotor influences as a result of toxaemias. The patient was treated for these toxic disturbances and her recovery has been most satisfactory.

While I hold the oculist and aurist in high esteem as specialists, the result of my observations has been to convince me that specializing has too sadly narrowed the field of medicine.

My sympathies have always been aroused by the victims of the patent-medicine sharks, who take advantage of the weakness of human nature, as expressed by the late P. T. Barnum, that "over ninety per cent of the people wish to be humbugged." It is a well-known fact that the people have been robbed of millions of dollars by these unscrupulous "fakers." I shall cite only one example of the many I know, viz.:

There are very many "sure-cure" patent remedies for rheumatism, and I believe I can truthfully say that not one of those who place such frauds before the people is aware of the etiological factors that enter into the disease which is called rheumatism. Even the most advanced scientific research has failed to solve this problem of nutritive disturbances. I believe, however, that with the education of the people there will come a day when the wisdom, integrity and capability of the medical profession will be adequately appreciated, and that the many cults which have so infamously deluded the public will meet with the destruction they so well deserve.

It is only of late years, and as a result of the advance in scientific laboratory work, that the practice of medicine has been classed as a science. From earliest times, however, the work of the physician, in his efforts to relieve suffering humanity, has been recognized as a noble art; and with the further advance of knowledge it is certain that the physician will be held in still higher esteem, if only the element of commercialism in the profession can be eliminated.

### SOME OBSERVATIONS IN REGARD TO INTESTINAL INFECTIONS.

The intestinal disturbances are so many and so varied, that any attempt to discuss this important subject would lead us into an endless labyrinth. Our research workers have lately been giving especial attention to this particular class of disturbances.

The prima via (alimentary canal), in its bearings on nutrition and disease, so invites the attention of the physician, that we must not overlook it. We will, in a limited degree, touch upon one form of dysentery, viz.: tropical dysentery, due to the motile amoebia, and its resulting infections. When it was demonstrated that the motile amoebia, though a wanderer, has its favorite habitat in the colon, the source of tropical dysentery and frequently the cause of much suffering and death, many of our most capable investigators devoted themselves to the study of this great disturber of the peace and welfare of mankind. The results achieved in this single field merit a monument to the efficiency and painstaking work of the physician. As a result of these investigations, it was demonstrated that the motile amoebia is the cause of varied and remote nutritive disturbances. When we contemplate the result of the investigations on this subject, we are appalled at the deadly effect of this single source of infection.

In the early days of my professional work in the swamps of Madison Parish, Louisiana, and elsewhere in the South, I had the advantage of observing under favorable circumstances the question of amoebic dysentery. I there learned the great importance of seeking information even from the humblest sources. Acting upon this principle, I was able to learn from the ignorant negro the method of obtaining the best results in the treatment of dysentery. Watching their methods, I observed that these negroes resorted to the forest for their medicament and that they obtained better results in meeting the ravages of bloody-flux than those who in much higher position financially and socially were able to obtain, despite the fact that they were able to command the aid of the best medical and hygienic conditions. Pursuing my investigations as based upon these observations, I found that the negroes had made use of crude turpentine boluses, and by the use of them had brought about favorable results in combating the causes of bloody-flux.

It was soon after this that the germ theory of diseases and the study of germicides gained prominence. It then became evident to me that turpentine as a germicide was an efficient factor in destroying not only the motile amoebia but also other deleterious germ organisms.

Oil of turpentine (oleo-terebinth), an essential oil, has the properties of a great antiseptic with a rapid diffusibility through the animal tissues. Throughout my varied experience, I have not known of a single instance where turpentine, when used properly, has caused the slightest injury, but, on the contrary, have known of many instances in which it has resulted beneficially. I will cite only one or two instances where turpentine was used by me with good results.

Captain F. L. N. of the U. S. M. Service, a man of about sixty years of age, was suffering from motile amoebia infection, probably contracted in the tropics in his early days. I had known the captain for many years and knew that he was subject to a torpor or constipation of the bowels. Twenty years before this time I had treated him for articular rheumatism and had told him that, in my judgment, his rheumatism was the result of an infection, probably from the intestinal tract, but that I was not able to identify the type of infection in order to properly treat it. Some few years ago the doctors of San Francisco were surprised to observe that amoebic dysentery seemed to have gained some foothold in the city, in spite of the fact that our particularly healthful climate would apparently make such a thing impossible. In combating the disease we were fortunate in having the efforts of our local pathologists supplemented by those of several very efficient physicians of U. S. M. Preventive and Protective Medicine, men who had had much experience in fighting tropical diseases in the Philippine Islands. Captain N. was referred to one of the ablest of these men. We were surprised to learn that amoebic infection had been discovered, with complications, especially that of chronic arthritis, which had reached a state of arthritis deformans. The U. S. M. physician had demonstrated that many cases of rheumatism were the result of amoebia, and that by overcoming this germ these rheumatic conditions were removed. The patient was treated for some time, but without result. About a year later the Captain returned to me for treatment, and, upon examination, I found that he was still infected with amoebia. In treating him I made use of turpentine, Ingol's Solution, etc., and succeeded in destroying the amoebia. Frequent examinations within the past two years, both by myself and other physicians, have disclosed that there has been no recurrence of the disease.

Another interesting case was that of Mr. F., a man of about forty-five years of age. He had been very ill from amoebia infection and its consequences, and had for some time been under the treatment of some of our ablest physicians. When he came under my care he was in a very serious condition. I found that his stools showed an abundance of motile amoebia and general disturbances of the system as sequelae. I used the same treatment as in the previous case, and within a short time the patient's health was thoroughly restored.

I could mention other cases of a similar character, but will simply say that through the knowledge gained from my observation of the negroes I have been successful with every case of amoebia dysentery that I have been called upon to treat.

In commenting upon the value of turpentine, I cannot refrain from relating an experience which I had early in my professional career.

In Georgetown, S. C., a patient of mine died from child-bed fever. At that time the medical profession was not aware of the important part played by septic organisms in this disease. My conscience was greatly troubled over the loss of this patient, for I felt that either the medical profession was very deficient or that I had mistaken my calling. I was very well acquainted with an elderly physician in the city who had told me that in his long experience he had never lost a case of puerperal septicaemia. I asked him how it was that, with so many cases of confinement, he had not lost one from child-bed fever. The doctor met my request for information with this explanation: "On the second day of all my confinement cases I give the patient a dose of 'half and half,' that is, a tablespoonful of turpentine with an equal quantity of castor oil. This does the work but I cannot say how." The "why" he gave me, the "how" I afterwards comprehended. I profited by this advice to such an extent that I have never had to combat a case of puerperal sepsis in my professional work. I also believe that I have saved many lives by the judicious use of oil of turpentine, etc., even when I have been called in consultation in puerperal sepsis and septive abortive cases.

The therapeutical value of oil of turpentine has not, in my estimation, received proper consideration as a germicide, either by the medical profession or by the laity.

My attention was once called by my preceptor, Dr. Simon Baruch of New York City, to a case where he had been called in consultation. The little patient was apparently dying from diphtheria, and, as a last resort, he suggested that the child be given a teaspoonful of oil of turpentine combined with, I believe, an equal quantity of castor oil. That night the nurse, by mistake, gave the child a dose of one ounce of turpentine instead of one drachm. When the physicians met next morning they found that the child's condition was very much improved, and subsequently a complete recovery was effected.

Not long after this, while on the Mallory Line steamer sailing from New York to Galveston, my son, a child between six and seven years of age, was stricken with a severe attack of diphtheria. As we were at sea it was difficult to procure the proper drugs, and I

was very much handicapped in my efforts to combat the disease. I was fortunate, however, in having with me some oil of turpentine, and this was used freely, with the result that the child entirely recovered.

I have so often found oil of turpentine useful in cases where the early symptoms indicated lockjaw (tetanus), that I now make it a practice to use oil of turpentine in combating tetanus.

I have recently been reliably informed that the practice of diluting and adulterating oil of turpentine has been carried to such an extent that this valuable drug is often rendered impure and unfit for use. It is extremely unfortunate that commercialism should be carried so far.

Before the value of antitoxin in the treatment of diphtheria had been ascertained, I relied on the use of oil of turpentine in my treatment of this disease, viz., diphtheria, with very gratifying results.

I consider that septic infections are the cause of most of our visceral diseases. For example, I have frequently seen cases in which the kidneys were affected and the cause had been attributed to the existence of Bright's disease. Upon careful examination it developed that the albumen and casts found in the urine were due to some remote source of infection, and that the apparent kidney trouble was merely a symptom. This has been repeatedly demonstrated in obscure cases of infected appendix. By looking to the appendix as a source of infection and removing the same, these kidney disturbances have been cleared up and the patient's health and nutrition fully restored.

I repeat that the physician cannot give too much consideration to the prima via as a source of infection. The term "appendix indigestion" is not far-fetched. While surgery is the most fascinating branch of our profession, I must lay stress upon my belief that if the physician, in this advanced age of pathology and etiology, will keep pace with the splendid results attained in combating disease, not only will the work of the surgeon be more successful but the necessity for surgical operations will be greatly lessened.

In conclusion, and as confirming the belief that unworthy practitioners have in many cases cast discredit upon the medical profession, I may refer to the book recently published by Mr. George Bernard Shaw entitled "The Doctor's Dilemma," in which he treats satirically, and in some respects contemptuously, of the work of the physician. The author thereby shows that the work of inefficient practitioners and of the followers of the various "cults" has reflected itself upon the general community to the detriment of the high reputation and esteem in which the medical profession should rightly be held.

